# Glucagon−like Peptide−1 and−2 Levels in Children with Diabetic Ketoacidosis

**DOI:** 10.4008/jcrpe.v1i3.12

**Published:** 2009-02-05

**Authors:** Ayşehan Akıncı, Özgür Aydın, Halil İbrahim Özerol

**Affiliations:** 1 Pediatric Endocrinology Department, İnönü University, Turgut Özal Medical Center, Malatya, Turkey; +90−422 341 06 60+90−422 341 07 28aakinci@inonu.edu.trPediatric Endocrinology Department, İnönü University, Turgut Özal Medical Center, Malatya, Turkey

**Keywords:** Glucagon−like peptide 1, glucagon−like peptide 2, diabetic ketoacidosis, type 1 diabetes mellitus

## Abstract

**Objective**: The aim of this study was to investigate whether insulin deficiency and increased catabolism may have a role in the regulation of plasma glucagon−like peptide (GLP)−1 and GLP−2 levels in children with diabetic ketoacidosis (DKA) and whether insulin treatment may affect the levels of these polypeptides.

**Methods**: Plasma GLP−1 and −2 levels were measured in 24 patients with DKA aged 8 to 14 years before insulin infusion (time 0), when ketonemia and acidosis disappeared (time 1), and when weight gain started (time 2). Eighteen healthy children aged 8 to 14 years constituted the control group.

**Results**: At time 0, mean plasma GLP−1 and GLP−2 levels were significantly elevated in the patients compared with the control group (p<0.05 and p<0.01, respectively). At time 1 when ketonemia and acidosis disappeared, GLP−1 and GLP−2 levels decreased significantly from the initial levels (p<0.05 and p<0.01, respectively). At this time, while GLP−1 level was not different from that of the controls, GLP−2 level was higher than that of the controls (p<0.05). GLP−1 and−2 levels did not show any significant differences between the patients and controls when weight gain started (time 2).

**Conclusion**: Our results show that DKA is associated with increased plasma GLP−1 and −2 concentrations. Effective fluid and insulin treatment resulted in a significant decrease in plasma GLP−1 and −2 levels. This may be due to the negative feedback effect of insulin on the production of these polypeptides.

**Conflict of interest:**None declared.

## INTRODUCTION

Glucagon−like peptide−1 (GLP−I) and GLP−2 are proglucagon−derived peptides produced in the L−cells of the small intestine and secreted into the circulation after food intake. GLP−1 stimulates insulin secretion in a glucose−dependent manner, and inhibits glucagon secretion, gastric motility and food intake.(1, 2, 3, 4, 5, 6, 7) Recent studies also reported that GLP−1 increases pancreatic b−cell mass by stimulating b−cell proliferation and inhibiting b− cell apoptosis.(8) GLP−1 administration to rodents and humans lowers plasma fasting and food−stimulated glycemia, and it has also been shown that GLP−1 induces satiety and reduces food intake in both healthy and diabetic subjects. GLP−2 does not seem to regulate blood glucose, but it has insulinotropic effects on the intestinal mucosa.(9, 10) It has been shown that GLP− 2 stimulates the proliferation of small intestine and large bowel mucosal epithelial cells, and inhibits apoptosis both in animals and humans.(11) GLP−2 also enhances intestinal barrier functions and stimulates intestinal hexose transport.(12, 14) The secretion of GLP−1, GLP−2 and the other proglucagon derived peptides into the circulation is stimulated by ingestion of carbohydrate, protein and fat. Carbohydrate ingestion is the best stimulus for GLP−1 secretion, in both animals and humans. Several neural and humoral factors, are responsible for the rapid increase in GLP−1 secretion.(15, 16, 17) Although insulin administration to diabetic rats decreases the circulating intestinal proglucagon derived peptides,(18) the potential inhibitory effect of insulin on the synthesis of GLP−1 and GLP−2 has not yet been demonstrated in humans. It has been reported that type 1 diabetes mellitus (T1DM) is associated with a decreased GLP− 1 level, whereas less is known about GLP−2 levels.

The aim of the study was to evaluate the changes of the plasma GLP−1 and GLP−2 levels before and during insulin treatment in diabetic ketoacidosis (DKA) which is a state characterized by extreme insulin deficiency. The study also aimed to investigate the confounding parameters in the secretion of these peptides.

## METHODS

The study included 24 patients (12 boys and 12 girls) with T1DM, aged 10.5±0.8 (mean±SD) years. The control group consisted of 18 healthy children (10 boys and 8 girls), aged 10.2±1.5 years. Patients were admitted to our hospital with a diagnosis of DKA (an arterial pH <7.3, glucose levels >300 mg/dL, and ketonemia). Only two subjects had new−onset T1DM. The mean duration of the diabetes was 25.8±4.5 months. Patients were treated with intravenous fluids and continuous insulin infusion according to the DKA treatment protocol. Sodium bicarbonate was administered to patients with an arterial pH<7.2. The total insulin dose used ranged from 0.52 to 1.41 U/kg over the first 24 hours. All patients were started on oral fluids and food approximately 9−12 hours after the start of the treatment. Blood was collected for the measurement of plasma GLP−1 and −2 three times: before starting insulin and fluid treatment (time 0), when ketonemia resolved (time 1) and when weight gain started (time 2), approximately 96 hours after the start of treatment. Blood was collected from the control group only once. Heights and weights were measured by standard methods and BMI (kg/m^2^) calculated and expressed as SDS according to national standards.

Detailed consent forms of the study were signed by the families of the patients and control group. Local committee approval was obtained for the study.

Biochemical analyses: Plasma glucose concentrations were measured using the glucose oxidase method. Plasma ketone bodies and glycated hemoglobin (HbA_1c_) levels were measured with enzymatic methods from venous blood samples. Arterial blood gases were measured with an automatic blood gas analyzer (Nova Biomedical pHOXplus L). For the measurement of plasma GLP−1, and GLP−2, blood samples were collected using a chilled syringe and transferred into a polypropylene tube containing EDTA (1 mg/ml of blood) and Aprotinin (500KIU/ml of blood) at 0°C. The blood sample was centrifuged at 1600 xg for 15 minutes at 0°, and transferred into a fresh polypropylene tube, and stored at –70°C. After an extraction procedure for plasma, GLP−1 levels were measured by ELISA with the reagents of Peninsula Laboratories, Inc. (San Carlos, California) with the detection range of 0−25 ng/ml for standard curve and GLP−2 levels were measured by RIA with the reagents of Peninsula Laboratories, Inc. (San Carlos, California) with the detection range of 10−1280 pg/100 μl for standard curve according to protocols that the producer company proposed.

Statistical analyses: These were performed using a statistical package (SPSS for Windows, version 11.01). Study and control group comparisons were made by the Mann− Whitney U−test. Intragroup comparisons were performed by using the Wilcoxon test. Spearman correlation analysis was used to investigate the degree of correlation between age, body weight, BMI, insulin dose, insulin injection number per day, arterial blood pH, glycemic control as reflected by HbA_1c_ value and plasma GLP−1 and GLP−2 concentrations. The data are expressed as means±SDs in the text. For all comparisons, statistical significance was defined as p< 0.05.

## RESULTS

**Figures 1:**
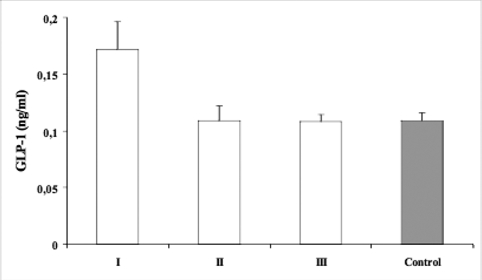
GLP−1 levels; before starting treatment (I) (time 0), when ketonemia resolved (II) (time 1) and when weight gain started (III) (time 2) in children with DKA and in control subjects.

GLP−1 and −2 levels of the study population are presented in Table 1. The insulin and fluid administration effectively decreased blood glucose levels and improved the metabolic disturbances. BMI and BMI SDS values in patients were significantly lower than those of the control subjects at admission (p<0.05, Table 1). HbA_1c_ (%) levels were significantly higher in patients (13.2 ± 4.1) compared with those of controls (4.1±0.3) (p<0.001). Mean arterial blood pH value was 7.14±0.04 in the patients. Plasma ketone bodies disappeared during the initial 6−8 hours, and arterial blood pH normalized within 2−4 hours of treatment. Before treatment, plasma GLP−1 and GLP−2 levels were significantly higher in patients than in controls (p<0.05 and p<0.01, respectively, Table 1, Figures 1 and 2). Plasma GLP−1 levels decreased significantly at 8th hour after starting treatment (time 1) when ketonemia resolved (p<0.05, Table 1). No significant difference was found between the patients and controls at this time (time 1) (Table 1, Figure 1). GLP−2 levels also showed a significant decline from time 0 to time 1 in the patient group (p<0.05). The level was significantly higher than in the controls (p<0.01, Table 1, Figure 2). Plasma GLP−1 and −2 levels further decreased at time 2 (at 96 hours of treatment) when weight gain started, and values were comparable to those of the control subjects (Table 1, Figures 1 and 2).

In the patient group, there were no significant correlations between age, body weight, BMI SDS, HbA_1c_, insulin dose and plasma GLP−1 and −2 levels except for a significant negative correlation between the GLP−2 levels and arterial pH values (r= –0.26, p<0.05).

**2:**
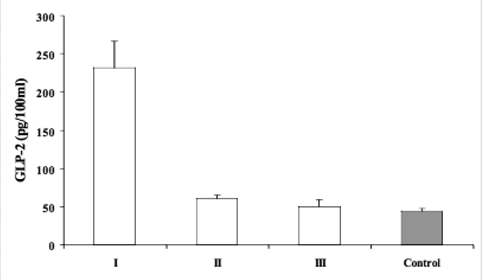
GLP−2 levels; before starting treatment (I) (time 0), when ketonemia resolved (II) (time 1) and when weight gain started (III) (time 2) in children with DKA and in control subjects.

**Table 1:**
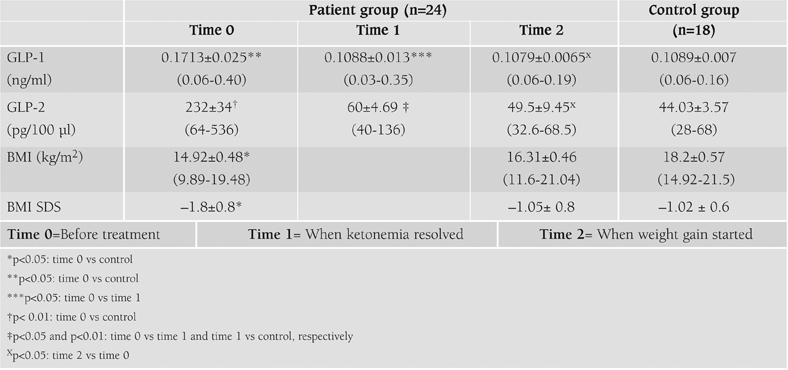
Plasma GLP−1 and −2 levels and BMI, BMI SDS values of the patients and controls.

## DISCUSSION

There are few studies about circulating GLP−1 and GLP−2 levels in patients with T1DM in the literature. Most of the studies were performed in type 2 diabetic patients (T2DM) and were based on GLP−1 response to nutrient intake.(19, 20, 21, 22, 23, 24) Some of these studies revealed that GLP−1 response to nutrient intake was reduced in patients with T2DM as compared to healthy controls,(21, 25) whereas in other studies it was found to be increased or not altered.(26, 27) In T1DM, preliminary studies have suggested that endogenous secretion of GLP−1 may be defective.(28) Other studies conducted among type 1 diabetics under good metabolic control and on intensive insulin regimen, fasting and postprandial GLP−1 concentrations were found to be near−normal or low, compared to healthy subjects, and the decreased levels were attributed to the existence of possible negative feed−back effect of insulin administrated exogenously.(22, 23, 24, 25)

Decreased blood glucose levels and also reduction of glucose excursion after meals were observed in recent−onset type 1 diabetic patients treated with intravenous or subcutaneous GLP−1 with concurrent administration of multiple dose insulin therapy. These effects of GLP−1 were attributed to inhibition of gastric emptying, enhancement of insulin secretion, and also decreased glucagon secretion.(9, 20, 22, 29, 30) Some studies, however, have reported negative results.(5, 31)

In the current study, we found elevation of plasma GLP−1 and GLP−2 levels before the start of DKA therapy. After starting treatment, GLP−1 and GLP−2 levels started to decrease in the first 8 hours during which none of the patients consumed any food. This result implies that food intake has no direct effect on the changes of GLP−1 and GLP−2 secretion in DKA. Thus, exogenous insulin infusion seems to have led to the decrease of plasma GLP−1 and GLP−2 levels in our patients. Furthermore, we did not detect any differences between plasma GLP− 1, and GLP−2 levels of the patients and controls when weight gain started. Taken together, our results suggest that insulin has an inhibitory effect on GLP−1 and−2 secretion and the glucose lowering actions of GLP−1 are regulated by insulin as reported before. Although proteins and fatty acids were found to stimulate or potentiate GLP−1 release in a number of studies,(32, 33, 34) the underlying mechanisms have not yet been clarified. Since the low−insulin catabolic process is prominent in DKA, it may be postulated that hyperglycemia and increased plasma free fatty acid (FFA) levels may cause GLP−1 release by stimulating the intestinal−L cells. Furthermore, decreased renal blood flux due to excessive fluid loss may be related to the elevation of plasma GLP−1 and GLP−2 levels in DKA.

A number of animal studies have described elevated levels of both plasma and tissue GLP−1 and GLP−2 in STZ−induced diabetic rats without insulin therapy.(35, 36, 37, 38) The elevated GLP−2 concentrations seen in STZ diabetes were prevented by treatment with exogenous insulin, and it has also been shown in animal studies that insulin suppressed the increase of plasma levels of GLPs as well as intestinal growth.(18) In our study, the high plasma GLP−2 in DKA can be ascribed to several factors, including insulin deficiency, increased stress hormones, accumulation of ketone bodies and dehydration.

It has been shown that plasma GLP−2 levels are elevated in the setting of intestinal injury models in animals and in humans.(39, 40) Acute abdominal pain, a common feature of DKA may develop due to mesenteric ischemia or to the irritative effect of ketone bodies and improves with resolution of the ketonemia after administration of fluids and insulin. Although mesenteric ischemia is uncommon in children, there are a few reports of pediatric patients with DKA who had mesenteric ischemia.(41, 42) In our study, none of the patients had abdominal complaints and none were diagnosed as mesenteric ischemia. However, non−occlusive bowel ischemia may occur as a result of severe mesenteric vasospasm which is a physiological response to hypovolemia and ineffective intestinal perfusion. In DKA, severe dehydration and decreased tissue perfusion associated with severe acidosis are not uncommon, so that the increased GLP−2 levels in DKA may be due to severe dehydration and acidosis which can lead to tissue hypoxia. The significant negative correlation between the GLP−2 levels and arterial pH values before treatment established in the present study supports this possibility.

In conclusion, in our study, the increased levels of GLP−1 and GLP−2 seem to be related to insulin deficiency in DKA. Mesenteric vasospasm which develops as a consequence of hypovolemia and severe acidosis may have contributed to the changes observed in the plasma GLP−2 levels in DKA. It is of interest that GLP−1 and GLP−2 levels decreased following insulin infusion, indicating the existence of a negative feed−back effect between these hormones.
